# Focally amplified lnc RNA genes in cancer

**DOI:** 10.18632/oncoscience.127

**Published:** 2015-02-18

**Authors:** Lu Yang, Xiaowen Hu, Xia Zhao, Lin Zhang

**Affiliations:** Department of Obstetrics and Gynecology, Perelman School of Medicine, University of Pennsylvania, Philadelphia, PA

Genomic instability could lead to the aberration of genes and partially be the cause of carcinogenesis. Researches of cancer drivers are mainly focused on protein-coding genes, especially those are harbored in the genomic regions with high frequent somatic copy-number alterations (SCNAs) in cancer. The protein-coding sequences only took up less than 2% of the whole human genome, although, nearly 30% was affected by SCNAs, leaving behind many “gene desert” altered regions in cancer genome. Recent findings revealed that noncoding RNAs made up the vast majority of transcribed RNAs. Long noncoding RNAs (lncRNAs) were defined as RNA transcripts without protein-coding function and with a length of more than 200 nucleotides. LncRNAs can be cell-type and tissue-specific, and the expression was developmentally regulated. By binding to RNA, DNA, or protein, lncRNAs may exert their biological functions, including cell proliferation, differentiation, apoptosis, immune response and migration, all of which had been implicated as both tumor suppressors and oncogenes. However, when we took a closer look at the protein-coding genes and lncRNAs inside the amplicon, challenge to identify a pivotal gene in tumorigenesis emerged. Furthermore, SCNAs of lncRNA genes contributing to cancer development remained to be elucidated.

In a recent study [1], we analyzed the single nucleotide polymorphism (SNP) arrays of 2,394 tumor specimens from 12 diverse cancer types, as well as the SCNA frequency of 13,870 lncRNA-containing locations. By integrating the gene expression microarrays of 40 established cancer cell lines, we found a set of oncogenic lncRNA candidates using all the three criteria listed below: copy-number gain was found in at least 25% of the samples in a single tumor type; lncRNA was mapped in a focally amplified region; the expression can be detected in more than half of the 40 cell lines. Next, we carried out short hairpin screening and successfully identified focally amplified lncRNA on chromosome 1 (FAL1), as a potential oncogenic lncRNA. Compared with hematologic and neural malignancies, FAL1 copy-number gain showed a significantly higher frequency in epithelial tumors. Though the RNA expression of FAL1 positively correlated with focal amplification, the phenomenon that some cell lines expressed high-level FAL1 RNA without genomic copy-number alterations was observed, suggesting other functional mechanisms. Further analysis of clinical ovarian cancer samples gave us a clear view that both RNA expression and genomic copy-number gain of FAL1 were higher in late-stage cancer and associated with decreased patients' survival. Several functional experiments were conducted to prove the oncogenicity of FAL1 besides the strong evidence of genetic analysis. Downregulation of FAL1 inhibited colony formation and cell growth, as well as the xenograft tumor growth, whereas overexpression of FAL1 promoted cell transformation, which can be enhanced by Myc or mutant Ras overexpression at the same time. Intriguingly, depletion of FAL1 had no effect on the expression of MCL1, a neighboring protein-coding gene located in the focal amplified region, showing an independent role of FAL1 when functioning. Rising to the challenge to explore the molecular mechanism of how FAL1 exerted the oncogenic activity, we proved that FAL1 physically associated with BMI1 protein, the core subunit of the chromatin-modifying polycomb repressive complex 1 (PRC1), and the vital binding domain with BMI1 was a 116 nt fragment in the middle of FAL1. The interaction sustained the stability of BMI1 and increased the ubiquitination level of H2AK119 and the activity of PRC1, which in turn altered the global transcriptional activities of PRC1 target genes. Among those transcripts regulated by FAL1 and BMI1, we identified cyclin-dependent kinase inhibitor 1A (CDKN1A), which encoded P21, had an impact on cell-cycle arrest and senescence, and at least in part demonstrated the oncogenicity of FAL1. Finally, intraperitoneal injection of FAL1 small interfering RNA remarkably inhibited tumor growth in an orthotopic mouse model of late-stage ovarian carcinoma, concomitant with upregulation of P21 protein levels.

In the aggregate, this work demonstrated the utility of an integrated method of bioinformatics and clinical information to systematically identify one single lncRNA, FAL1, with oncogenic activity. The functional interaction between FAL1 and BMI1 led us to the insight of molecular mechanism of lncRNA oncogenicity. Based on the fact that expression of lncRNAs trended to be cell-type and tissue-specific, FAL1 may be considerably beneficial as an informative biomarker and therapeutic target for cancer treatment.
